# Novel *USP9X* variants in two patients with X-linked intellectual disability

**DOI:** 10.1038/s41439-019-0081-7

**Published:** 2019-10-21

**Authors:** Yoshinori Tsurusaki, Yukiko Kuroda, Yasuko Yamanouchi, Eisuke Kondo, Kazunobu Ouchi, Yuichi Kimura, Yumi Enomoto, Noriko Aida, Mitsuo Masuno, Kenji Kurosawa

**Affiliations:** 1 Clinical Research Institute, Kanagawa Children’s Medical Center, Yokohama, Japan; 2 Faculty of Nutritional Science, Sagami Women’s University, Sagamihara, Japan; 3 Division of Medical Genetics, Kanagawa Children’s Medical Center, Yokohama, Japan; 4 Genetic Counseling Program, Graduate School of Health and Welfare, Kawasaki University of Medical Welfare, Kurashiki, Japan; 5 Department of Pediatrics, Kawasaki Medical School, Kurashiki, Japan; 6 Department of Radiology, Kanagawa Children’s Medical Center, Yokohama, Japan

**Keywords:** Genetics research, Genetic testing

## Abstract

*USP9X* variants have been reported in patients with X-linked intellectual disability. Here, we report two female patients with intellectual disability and pigment abnormalities along Blaschko lines. Targeted resequencing identified two novel heterozygous variants, c.4068_4072del (p. (Leu1357Tyrfs*12)) and c.1201C>T (p. (Arg401*)), in *USP9X*. Our findings provide further evidence that *USP9X* variants cause intellectual disability.

Intellectual disability (ID) is characterized by significant limitations in both intellectual functioning and adaptive behavior, with onset before the age of 18 years, and is commonly defined by an IQ score of <70^1,2^. ID occurs in ~1–3% of the population worldwide^1^. To date, almost 100 causative genes have been reported for X-linked ID (XLID)^3^.

Variants in *USP9X*, which is located at Xp11.4, have been suggested to cause various types of cancer, female-restricted X-linked syndromic mental retardation-99 (MIM 300968), and X-linked recessive mental retardation-99 (MIM 300919). *USP9X* encodes ubiquitin-specific protease 9×, which is highly expressed in the mouse brain and plays important roles in nervous system development, stabilization of myeloid leukemia cell differentiation protein (MCL1) in human follicular lymphomas and diffuse large B-cell lymphomas, and tumor cell survival^4–6^.

In this study, we identified novel *USP9X* variants in two female patients with XLID by using targeted resequencing.

Patient 1 is a 4-year-old girl who is the second child of healthy and nonconsanguineous parents. She was delivered at 37 weeks of gestation with a birth weight of 2278 g (−1.1 SD), a length of 47 cm (−0.1 SD), and an occipital frontal circumference of 30.5 cm (−1.5 SD). She was able to walk unsupported by 23 months. She began speaking in recognizable words at 2 years of age, at which point she also began to show signs of moderate-to-severe ID. The patient was referred to Kanagawa Children’s Medical Center because of developmental delay at 2 years of age. At the time of referral, she was 82.7-cm tall (−1.0 SD), weighed 13.4 kg (1.6 SD), and had an occipital frontal circumference of 46.4 cm (−0.7 SD). She had dysmorphic features consisting of upswept and curly hair, facial asymmetry, prominent forehead, bitemporal narrowing, short palpebral fissures, prominent nose with flared ala nasi, smooth philtrum, thin upper lip, full cheeks, dysplastic ears, tapering fingers, and Blaschko lines (Fig. 1a, b). She spoke several individual words and exhibited autistic behavior, including repetitive and stereotyped movements. At 3 years and 11 months of age, she developed obesity. She was 98-cm (0 SD) tall and weighed 18.6 kg (1.9 SD), and her body mass index was 19.4, which is >97th percentile for age. Her karyotype is 46,XX.

**Fig. 1:**
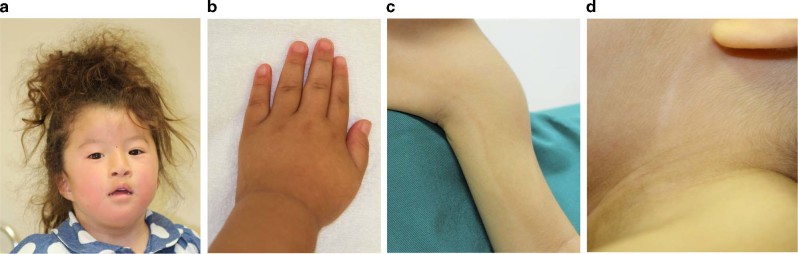
Patients with *USP9X* variants. **a** Patient 1 at 4 years of age. The patient exhibited upswept and curly hair, facial asymmetry, prominent forehead, bitemporal narrowing, short palpebral fissures, prominent nose with flared ala nasi, smooth philtrum, thin upper lip, full cheeks, and dysplastic ears. **b** Patient 1 at 4 years of age. The patient had tapered fingers. **c** Patient 2 at 18 months of age. Pigment changes along Blaschko lines bilaterally on the upper arms (**d**) and neck

Patient 2 is the first child of a 40-year-old primigravid mother and a nonconsanguineous 41-year-old father. Pregnancy was achieved by artificial insemination by using the husband’s sperm, and labor and delivery were uneventful. Owing to the advanced maternal age, prenatal cytogenetic analysis by using G-banding was performed on amniocentesis, and the results were normal. The maternal grandfather’s sister had a history of retinitis pigmentosa. The proposita was born at 41 weeks and 1 day of gestation. The newborn had a birth weight of 2968 g (−0.5 SD), a length of 48.3 cm (−0.9 SD), and a head circumference of 34.2 cm (0.4 SD). Apgar scores were 8 at 1 min and 9 at 5 min. During the early neonatal period, bilateral cryptotia, congenital hearing impairment as detected by auditory brain-stem response (rt-70 dB, lt-50 dB), and right muscular torticollis were noted. Bilateral clasped thumbs were identified at 2 months of age and had improved by 11 months of age. She was first evaluated by Kawasaki Medical School Hospital at 9 months of age because of developmental delay. The main clinical manifestations included epicanthus, telecanthus, short columella, depressed nasal tip, bilateral low-set and posteriorly rotated ears, overfolded helices, bifid uvula, umbilical hernia, and bilateral overlapping toes (T2–3, T4–5). At the time of evaluation, she was 73.4 cm (1.4 SD) in height, weighed 8465 g (0.3 SD), and had a head circumference of 44.8 cm (0.8 SD). Her muscle tone was normal. Pigment changes along Blaschko lines appeared bilaterally on the upper arms at 12 months and on the neck at 18 months (Fig. 1c, d). She suffered from recurrent otitis media with effusion after 12 months of age. She was able to lift her head at 4 months of age, sat alone at 8 months, babbled at 12 months, and walked unaided at 17 months. Her developmental quotient was 71 at 17 months. At 18 months, her height was 80.8 cm (0.5 SD), and her weight was 9.9 kg (0 SD). Ophthalmological evaluations revealed astigmatism, hyperopia, and normal fundi. Ultrasonographic examination of the heart and abdomen showed normal findings. Other normal laboratory tests included thyroid function (free T4, free T3), somatomedin-C, serum chemistries, immunoglobulins, complete blood counts, blood gas analysis, and urinalysis.

Clinical information was obtained after obtaining written informed consent from the patients’ families. The institutional review board of Kanagawa Children’s Medical Center approved this study. Genomic DNA was extracted from both patients’ peripheral blood by using the QIAcube (QIAGEN, Hilden, Germany) according to the manufacturer’s instructions. Targeted resequencing was performed for the two affected patients. Genomic DNA was captured by the TruSight One Sequencing Panel (Illumina, Inc., San Diego, CA, USA) and was sequenced on a MiSeq platform (Illumina) with 151-bp paired-end reads, as previously described^7^. The candidate variant was confirmed by Sanger sequencing.

Targeted resequencing identified two heterozygous variants in *USP9X* (NM_001039590.2): c.4068_4072del (p.(Leu1357Tyrfs*12)) in patient 1 and c.1201C>T (p.(Arg401*)) in patient 2. These variants were not present in the NHLBI-Exome Sequencing Project 6500, the 1000 Genomes Project, dbSNP138, the Human Genetic Variation Database or in our in-house Japanese exome database. Sanger sequencing confirmed that these variants had occurred de novo (Fig. 2).

**Fig. 2:**
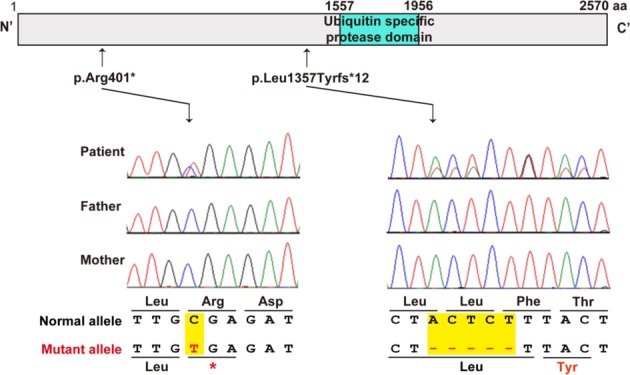
*USP9X* variants. Novel heterozygous variants identified in two female patients. USP9X is predicted to contain a ubiquitin-specific protease domain, as determined by SMART (http://smart.embl-heidelberg.de/). Electropherogram for each patient and her parents

Here, we report two female patients with heterozygous variants in *USP9X* who exhibited ID and pigment abnormalities along Blaschko lines (Supplementary Table S1). These two *USP9X* variants are truncating variants, resulting in a premature stop codon (Fig. 2). Reijnders et al. reported de novo loss-of-function variants of *USP9X* in 17 female patients^8^. Au et al. also reported de novo pericentric inversion resulting in a 0.326-Mb deletion of the *USP9X* 5′ UTR and a de novo truncating variant in two female patients^9^. Homan et al. reported two maternally inherited missense variants and a truncating variant of *USP9X* in three male patients^10^. This truncating variant is located in the last exon, and as such, the mRNA presumably escapes nonsense-mediated decay. Thus, USP9X alterations in female-restricted X-linked syndromic mental retardation-99 may confer loss-of-function effects. On the other hand, USP9X alterations in X-linked recessive mental retardation-99 may confer hypomorphic or a milder form of loss-of-function effects. The clinical features of our two patients were compared with previously reported female patients with variants in *USP9X* (Supplementary Table S1). All patients with *USP9X* variants exhibited ID. Some patients with ID also showed pigment abnormalities along Blaschko lines, including our patients. Dental abnormalities, asymmetric hypomastia, heart defects, urogenital abnormalities, scoliosis, postaxial polydactyly, seizures, hypotonia, and recurrent respiratory tract infections were observed in some of the previously reported patients but not in our patients. Further analysis is required to determine the phenotype–genotype correlation.

Endogenous USP9X localizes to the primary cilium; however, this localization was significantly decreased when USP9X expression was knocked down in fibroblasts by using siRNA^8^. Female heterozygous knockout mice (*Nes*-*Usp9x*^*−/X*^) were normal at birth and survived to adulthood^6^. A small reduction in the hippocampal area was observed in adult female knockout mice (*Emx1*-*Usp9x*^*−/X*^)^6^. However, no reduction in the hippocampal area was observed in our patients. In contrast, male hemizygous knockout mice (*Nes*-*Usp9x* ^*−/Y*^) died within 24 h of birth^6^.

In conclusion, we identified heterozygous *USP9X* variants in two female patients. Our report provides further evidence that *USP9X* variants are associated with XLID.

## Supplementary information

Supplementary Table S1

## Data Availability

The relevant data from this Data Report are hosted at the Human Genome Variation Database at 10.6084/m9.figshare.hgv.2621, 10.6084/m9.figshare.hgv.2624.
